# Use of a Fluorescent Aptamer RNA as an Exonic Sequence to Analyze Self-Splicing Ability of a Group I Intron from Structured RNAs

**DOI:** 10.3390/biology5040043

**Published:** 2016-11-17

**Authors:** Airi Furukawa, Takahiro Tanaka, Hiroyuki Furuta, Shigeyoshi Matsumura, Yoshiya Ikawa

**Affiliations:** 1 Department of Chemistry, Graduate School of Science and Engineering, University of Toyama, Gofuku 3190, Toyama 930-8555, Japan; apandaorange1@yahoo.co.jp (A.F.); smatsumu@sci.u-toyama.ac.jp (S.M.); 2 Department of Chemistry and Biochemistry, Graduate School of Engineering, Kyushu University, Moto-oka 744, Nishi-ku, Fukuoka 819-0395, Japan; t.tanaka.075@s.kyushu-u.ac.jp (T.T.); hfuruta@cstf.kyushu-u.ac.jp (H.F.)

**Keywords:** intron, ribozymes, self-splicing, spinach RNA, *Tetrahymena*

## Abstract

Group I self-splicing intron constitutes an important class of functional RNA molecules that can promote chemical transformation. Although the fundamental mechanism of the auto-excision from its precursor RNA has been established, convenient assay systems for its splicing activity are still useful for a further understanding of its detailed mechanism and of its application. Because some host RNA sequences, to which group I introns inserted form stable three-dimensional (3D) structures, the effects of the 3D structures of exonic elements on the splicing efficiency of group I introns are important but not a fully investigated issue. We developed an assay system for group I intron self-splicing by employing a fluorescent aptamer RNA (spinach RNA) as a model exonic sequence inserted by the *Tetrahymena* group I intron. We investigated self-splicing of the intron from spinach RNA, serving as a model exonic sequence with a 3D structure.

## 1. Introduction

The promotion of chemical transformation in a substrate-specific manner is one of the most important biological functions of naturally occurring biopolymers. In current cellular systems, protein enzymes are predominantly employed for such tasks. On the other hand, RNA molecules also conduct biologically important chemical transformations [1]. RNA molecules possessing enzyme-like functions are called RNA enzymes or ribozymes.

RNA splicing is an important step in a series of RNA processing events [2]. In RNA splicing, two RNA molecules (excised intron and ligated exons) are produced as the result of rearrangement of three RNA regions (5′-exon, intron, and 3′-exon) in the precursor RNA. The intron, which is present as the middle segment of the precursor RNA, is excised and the remaining two (5′- and 3′-exonic) segments are joined together to yield ligated exons as a new RNA fragment. Among the four distinct mechanisms utilized for RNA splicing [3,4], three have been shown to be supported by RNA-based machinery, in which RNA molecules are directly responsible for promoting sequential cleavage and the joining of phosphodiester bonds at particular positions (5′- and 3′-exon–intron boundaries) in RNA molecules [4]. Group I introns, which constitute one among three distinct classes of RNA-based splicing machinery, is excised from the precursor RNA by its own enzyme-like ability in a process called self-splicing [3]. The molecular mechanism and structural basis of group I intron self-splicing have been established, although several points, including structural rearrangement coordinating the first (cleavage at the 5’-exon/intron junction) and second steps (exon ligation) of the self-splicing, still have not been fully determined at an atomic level [3,5,6,7,8].

**Figure 1:**
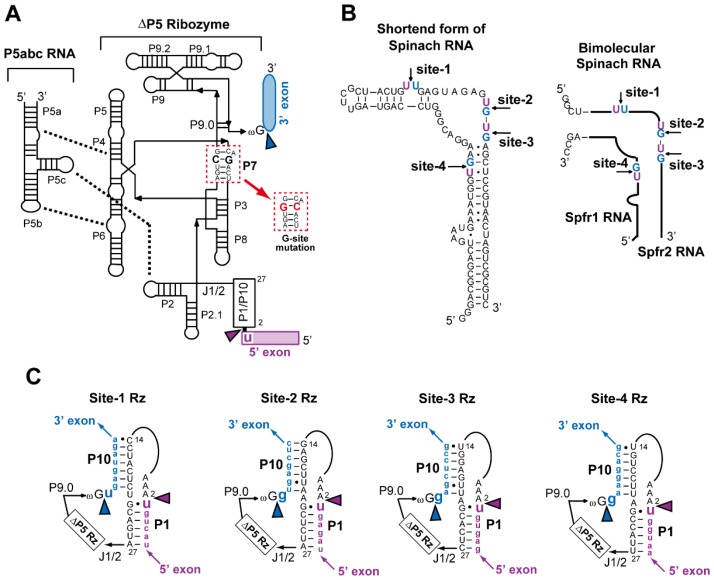
Secondary structures of the ∆P5 ribozyme/P5abc RNA complex (bimolecular derivative of the *Tetrahymena* intron) and spinach RNA employed as a model exonic sequence. (**A**) Secondary structures of P5abc RNA and ∆P5 ribozyme. Thick broken lines indicate the three tertiary interactions in assembly of P5abc and ∆P5 modules. Blue and purple arrowheads indicate 5′- and 3′-splice sites, respectively. (**B**) Nucleotide sequence of a shortened form of spinach RNA and schematic structure of its bimolecular derivative. Arrows indicate the sites where the ∆P5 ribozyme was inserted as a group I intron. (**C**) Sequences of P1/P10 elements of the four ∆P5 ribozymes. In each ribozyme, nucleotide sequences different from the wild-type *Tetrahymena* ribozyme are shown. The nucleotide sequence at positions 5–13 was the same as that of the wild-type ribozyme (5′-UAGCAAUAU-3′).

Self-splicing of group I intron RNA depends on a complex three-dimensional (3D) structure of the conserved core region of this intron class [5,6,7,8,9,10,11]. The core 3D structure of group I intron RNA has evolved to precisely recognize two exon/intron boundaries, at which two sequential cleavage/joining reactions proceed smoothly. In the sequential reactions, the transition state at each chemical step is effectively stabilized by the catalytic center organized within the core region of the intron RNA [9,10,11]. The catalytic center directly performing these roles is located within and around the P7 element (Figure 1A), which not only coordinates at least two catalytic Mg^2+^ ions [12] but also provides a specific recognition site (G-site) for guanosine [13,14], the recognition of which is essential for both splicing steps. Group I introns are found as intervening sequences in a variety of RNA sequences, the majority of which are non- or loosely structured mRNAs. Some group I introns, however, are found within noncoding RNAs, such as tRNAs and ribosomal RNAs [15]. These noncoding RNAs exhibit their functions through folding into defined 3D structures, meaning that, in their precursor RNAs, both intronic and exonic sequences potentially fold into 3D structures.

In the biotechnological application of self-splicing and its related reactions of group I introns, methods for artificial insertion of introns into RNA sequences of interest are necessary [16,17,18,19,20,21,22,23]. Regardless of structured or unstructured RNAs, the identification of insertion sites suitable for group I intron splicing is a key issue. In the case of mRNAs as exonic sequences, several studies have identified insertion sites through rational or screening methods [16,17,18,19,20,21,22,23]. In contrast to long RNA sequences as exonic sequences, both naturally occurring examples and the artificial engineering of group I introns, which are inserted into small and 3D-structured RNAs, such as tRNAs and aptamer RNAs, are limited. The development of model splicing systems is useful to analyze self-splicing of group I introns from short RNAs with 3D structures. In this study, we employed spinach RNA as a model exonic sequence that is both short and highly structured. Spinach RNA acts as an aptamer to recognize 3,5-difluoro-4-hydroxybenzylidene imidazolinone (DFHBI), which exhibits fluorescence only in a complex with spinach RNA [24,25,26]. This property is useful for monitoring the self-splicing activity of group I introns because the production of ligated exons can be traced with fluorescence in a solution containing DFHBI. As a model group I intron ribozyme inserted into spinach RNA, we employed a bimolecular version of the *Tetrahymena* intron ribozyme [27], because its splicing reaction can be easily controlled through reconstitution.

## 2. Materials and Methods

### 2.1. Plasmid Construction and RNA Preparation

Plasmids encoding precursor RNAs were derived from pTZ∆P5 [27], which encodes ∆P5 mutant of the *Tetrahymena* group I intron ribozyme. These plasmids were constructed by PCR-based mutagenesis. The sequences of the constructed plasmids were confirmed using a 4300 DNA analyzer (Li-COR, Lincoln, NE, USA). Precursor RNAs and P5abc RNA were prepared via in vitro transcription with T7 RNA polymerase and PCR-amplified DNA templates. The T7 promoter sequence was attached via PCR with the T7 promoter-containing sense primer. In vitro transcription reactions of ∆P5 ribozyme precursors were performed in the presence of 5 mM Mg^2+^ ions and 4 mM rNTPs. Transcription of P5abc RNA was performed with 15 mM Mg^2+^ ions and 4 mM rNTPs. Each transcription reaction was performed for 4.5 h at 37 °C. The DNA template in the reaction mixture was degraded using DNase I. Crude transcript was purified on a 4% or 6% denaturing polyacrylamide gel. 3′-End labeling of purified RNAs with the BODIPY fluorophore was carried out according to a previously reported procedure [28].

### 2.2. In Vitro Self-Splicing Assay with Denaturing Polyacrylamide Gel Electrophoresis

The BODIPY fluorophore-labeled precursor RNA (5.0 pmol: final 250 nM) and P5abc RNA (7.5 pmol: final 375 nM) were incubated separately at 80 °C for 5 min. Precursor RNA and P5abc RNA were then mixed at 37 °C and incubated for 30 min. After incubation of the resulting solution, the reaction was started by adding a 5× folding buffer (final concentration: 30 mM Tris-HCl, pH 7.5, 5 mM MgCl_2_, and GTP 0.2 mM) and incubated at 37 °C. At each time point, 4 μL of the solution was taken and mixed with 4 μL of the stop solution containing 75% formamide, 100 mM EDTA, and 0.1% xylene cyanol. The mixtures were separated on a 15% denaturing polyacrylamide gel. The gel was analyzed with a FluoroImager Pharos FX (Bio-Rad, Hercules, CA, USA). Product yields were calculated by the following equation: product yield (%) = 100 × [intensity of ligated exons]/[intensity of precursor RNA + intensity ligated exons].

### 2.3. In Vitro Self-Splicing Monitored by Fluorescence of Spinach RNA–DFHBI

**Figure 2:**
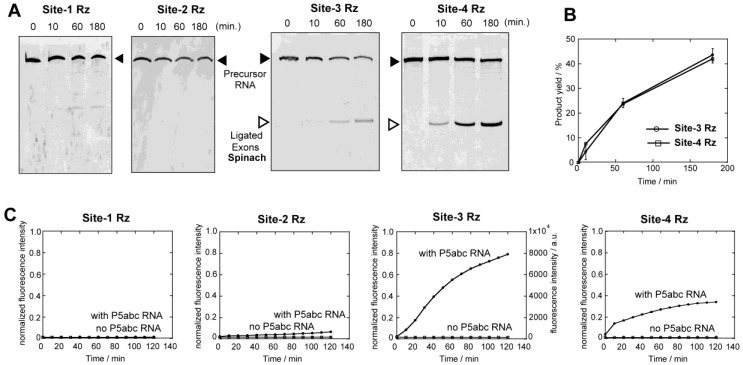
In vitro splicing of the ∆P5 ribozyme inserted at four different positions in spinach RNA with or without an assistance of P5abc RNA. (**A**) Denaturing polyacrylamide gel images of splicing reactions. (**B**) Time courses of the production of ligated exons (spinach RNA) calculated from Figure 2A and its duplicate assays. (**C**) Time-dependent increases in fluorescence of spinach RNA–DFHBI complex. Spinach RNA was produced as ligated exons. Fluorescence intensities were normalized (see Materials and Methods). Fluorescence of the site-3 ribozyme was monitored with a 40 µL solution so that this data can be compared directly with those in Figure 3, Figure 5D and Figure 6B.

Precursor RNA (final concentration: 0.60 μM) and P5abc RNA (final concentration: 0.88 μM) were incubated separately at 80 °C for 5 min. Precursor RNA and P5abc RNA was then mixed at 37 °C and incubated for 30 min. To this solution (45 μL), a 10× reaction buffer (5 μL, final concentration: 30 mM Tris-HCl, pH 7.5, 5 mM MgCl_2_, 125 mM KCl, and 100 μM DFHBI) were mixed to prepare a 50 μL reaction solution. An appropriate volume (40 μL or 50 μL) of the solution was transferred to a microplate well with a mineral oil overlay. The plate was then incubated at 37 °C for 120 min in a plate reader (Infinite F200 Pro; Tecan, Männedorf, Switzerland), which was pre-warmed at 37 °C. Fluorescence intensity was measured every 10 min. In an assay of bimolecular spinach aptamer, the partner RNA fragment (final concentration: 0.60 μM) of the splicing product was added to the reaction mixture. To compare the reactions with different solution volumes containing different amounts of RNA and DFHBI, raw values of fluorescence intensity were divided by the solution volume of the reaction mixture. The resulting values were then employed to derive normalized fluorescent intensities shown as vertical lines at the left side (Figure 2C).

### 2.4. Cotranscriptional Self-Splicing Assay

Template DNA (2000 ng), purified P5abc RNA (final concentration: 1.1 μM), a 10× transcription/splicing buffer (final concentration: 40 mM Tris-HCl, pH 7.5, 15 mM MgCl_2_, 125 mM KCl, 10 mM DTT, 2 mM spermidine, and 100 μM DFHBI), a 10× rNTP solution (final concentration: 1 mM each), a recombinant T7 RNA polymerase, and a recombinant RNase inhibitor were mixed to prepare a 50 μL reaction solution. Aliquots of 40 μL of the solution were quickly transferred to a microplate well, with a mineral oil overlay. The resulting plate was incubated at 37 °C for 120 min in the plate reader (Infinite F200 Pro; Tecan) that was pre-warmed at 37 °C. Fluorescence intensity was measured every 10 min.

### 2.5. Electrophoretic Mobility Shift (EMS) Assay

Unlabeled precursor RNA (1.0 μM) and BODIPY fluorophore-labeled P5abc RNA (250 nM) were incubated separately at 80 °C for 5 min. The two RNA solutions were mixed, and the resulting solution was incubated at 37 °C for 5 min. The 10× folding buffer (final concentrations: 71.2 mM Tris-borate, pH 8.3, and 0 mM, 5 mM, or 15 mM Mg(OAc)_2_) was added to the solution. The mixture was incubated at 37 °C for 30 min and subsequently incubated at 4 °C for 30 min. After adding 1/6 volume of glycerol containing xylene cyanol (0.1%), the samples were loaded onto a 5% nondenaturing polyacrylamide gel (29:1 acrylamide/bisacrylamide) containing 71.2 mM Tris-borate (pH 8.3) and 5 mM or 15 mM Mg(OAc)_2_ or 0.10 mM EDTA. Electrophoresis was performed at 4 °C, 200 V for the initial 5 minutes, followed by 75 V for 4.5 h. The resulting gel was analyzed with a FluoroImager Pharos FX (Bio-Rad).

## 3. Results

### 3.1. Experimental Design

To determine the effects of RNA secondary and 3D structures in exonic sequences on self-splicing of the *Tetrahymena* intron ribozyme, we inserted the intron ribozyme at four different positions in the secondary structure of a shortened derivative of spinach RNA (Figure 1B, left). To enable the *Tetrahymena* ribozyme to be spliced out, this ribozyme requires the correct formation of P1 substrate duplex for the first step of self-splicing. After the first step of splicing, a local structural change must occur in the intermediate RNA to form a P10 duplex [29], which is essential for the second step of splicing. To fulfill these requirements in each of the four positions serving as exon–exon junctions, we modified 14 nucleotides (positions 14–27) of the ribozyme to form correct P1 and P10 base pairs with given exonic sequences (Figure 1C).

The parent *Tetrahymena* ribozyme exhibits high self-splicing activity due to the high stability of its catalytically proficient 3D structure. While high self-splicing activity is advantageous in application of the intron ribozyme, this property also makes the intron difficult to analyze biochemically because the precursor RNA possessing 5′- and 3′-exons self-splices rapidly during in vitro transcription. To avoid the difficulty in preparing the precursor RNA, we used a derivative of the *Tetrahymena* intron that was dissected into the ∆P5 ribozyme module and the P5abc activator module (Figure 1A). The ∆P5 ribozyme contains all components involved directly in the catalytic mechanism, and is active in buffers containing high concentrations of Mg^2+^ ions (>15 mM) because Mg^2+^ ions can generally stabilize RNA tertiary structures [30,31]. On the other hand, ∆P5 ribozyme is nearly inactive under conditions with a low concentration of Mg^2+^ ions (<10 mM) due to the insufficient stability of its active tertiary structure [32]. The precursor RNA of ∆P5 ribozyme, therefore, can be readily prepared by in vitro transcription with 5 mM Mg^2+^ ions. The P5abc module serves as an activator of ∆P5 ribozyme because the P5abc module associates tightly with the active structure of ∆P5 ribozyme [27,33,34]. The resulting ∆P5/P5abc bimolecular ribozyme is as active as the parent ribozyme possessing P5abc as a *cis*-acting module [33,34].

Based on the experimental design using ∆P5 intron ribozyme, the splicing efficiencies of four precursors were analyzed via two methods, which are an analysis of splicing products with denaturing polyacrylamide gel electrophoresis (denaturing PAGE) and a direct observation of fluorescence of DFHBI complexed with spinach RNA as an indicator of the product (ligated exons).

### 3.2. Analyses of Self-Splicing to Produce the Fluorescent RNA Aptamer

We first examined self-splicing reactions of four precursor RNAs in the presence of 5 mM Mg^2+^ ions by denaturing PAGE (Figure 2A,B). Among four precursors, only site-3 and site-4 ribozymes exhibited detectable activity to produce a band corresponding to ligated exons (Figure 2A). Production of the correct ligated exons was additionally confirmed by tracking the spinach RNA–DFHBI complex in the reaction solution (Figure 2C). A time-dependent increase in the fluorescence of the spinach RNA–DFHBI complex was observed in the reaction mixtures of the site-3 and site-4 ribozymes, but no emission was detected in the reaction of the site-1 and site-2 ribozymes (Figure 2C). Emission from the site-3 and site-4 ribozymes was not detected in the absence of P5abc RNA, indicating that the reactions are dependent on the reconstitution of the full-length ribozyme structure (Figure 2C). Site-4 ribozyme exhibited similar activity to site-3 ribozyme in the denaturing PAGE assay (Figure 2B) but was less active than site-3 in the fluorescence monitoring of the ligated exons (Figure 2C). This difference may be related to post-splicing events (disassembly between the ligated exons and the excised intron and folding of the ligated exons) required only for the formation of the spinach RNA (ligated exons)–DFHBI complex (Figure 2C).

**Figure 3:**
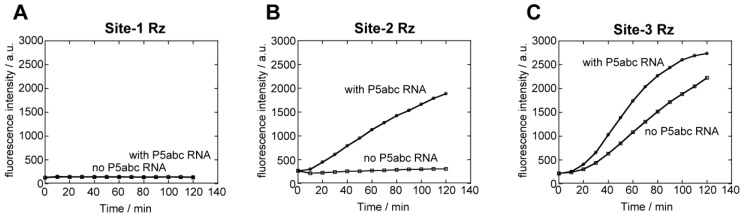
Cotranscriptional folding and splicing of the ∆P5 ribozyme inserted at site-1, -2, and -3 in spinach RNA with or without assistance of P5abc RNA. Separately prepared P5abc RNA was added to transcription solutions. Fluorescence was monitored with a 40 µL solution. Fluorescence of the transcription mixtures producing the site-1 ribozyme (**A**), the site-2 ribozyme (**B**), and the site-3 ribozyme (**C**).

To determine whether the splicing deficiencies of the site-1 and site-2 ribozymes are due to RNA folding conducted using a thermal denature/renature protocol, we then examined the splicing reactions of site-1, -2, and -3 precursor RNAs in a cotranscriptional manner (Figure 3). The transcription solution for T7 RNA polymerase was prepared with 15 mM Mg^2+^ ions and 4 mM nucleotide triphosphates, which can coordinate Mg^2+^ ions. In the presence of 1.1 μM P5abc RNA and the components needed for spinach RNA to emit fluorescence (125 mM KCl and 10 μM DFHBI), the transcription of the precursor of the site-3 ribozyme efficiently showed the fluorescence emission of spinach RNA–DFHBI complex (Figure 3C). Weak fluorescence emission was detected in the transcription mixture without P5abc RNA (Figure 3C). The cotranscriptional splicing was also performed with the precursor of the site-2 ribozyme. Cotranscriptional splicing of the site-2 ribozyme proceeded in the presence of P5abc RNA, yielding the fluorescence emission of spinach RNA–DFHBI complex (Figure 3B). However, fluorescence was not observed without P5abc RNA (Figure 3B). Regardless of the presence of P5abc RNA, no fluorescence was detected in the cotranscriptional reactions of the precursor of the site-1 ribozyme (Figure 3A).

Comparative analysis of precursor RNAs of the site-1, site-2 and site-3 ribozymes revealed their distinct splicing abilities between in vitro splicing of purified RNAs (Figure 2) and cotranscriptional splicing of the nascent transcript (Figure 3). As the three RNAs share the identical nucleotide sequence in the whole ribozyme except the exon-recognition elements (P1/P10 in Figure 1C), differences in splicing ability must be due to their RNA folding to produce catalytically active 3D structures.

### 3.3. Tertiary Folding of ∆P5 Ribozyme Probed by an Electrophoretic Mobility Shift (EMS) Assay with P5abc RNA

**Figure 4:**
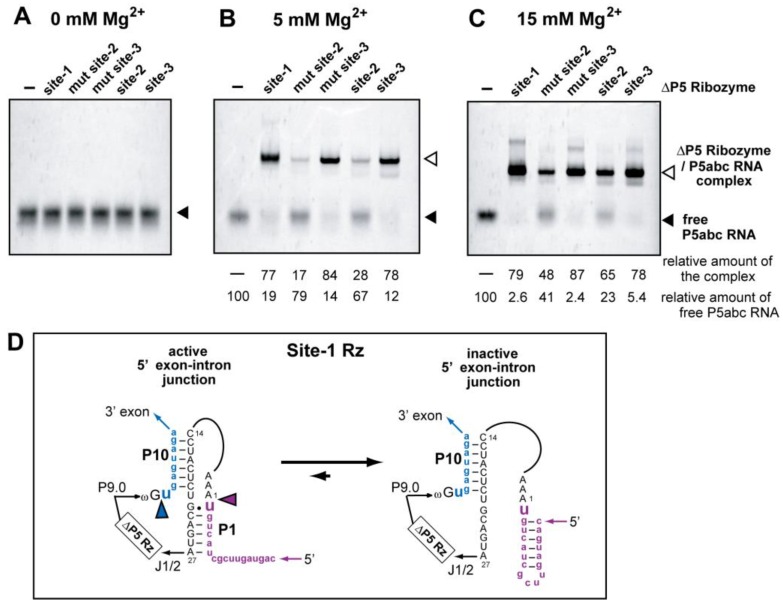
Electrophoretic mobility shift (EMS) assay of ∆P5 ribozyme/P5abc RNA complexes. (**A**–**C**) Mut site-2 and mut site-3 are mutants of site-2 and site-3 ribozymes, respectively, the P7 element of which possesses a G-C to C-G mutation at the guanosine binding site (G-site). P5abc RNA was 3′-end labeled with BODIPY fluorophore. (**D**) Two possible secondary structures of the 5′exon–intron junction in the site-1 ribozyme. The inactive form (right) is predicted to be more stable than the active form (left).

To probe the tertiary folding of ∆P5 ribozyme within their precursor RNAs, we performed an electrophoretic mobility shift (EMS) assay of the ∆P5/P5abc bimolecular complex (Figure 4). As P5abc RNA selectively recognizes and associates with the correctly folded ∆P5 ribozyme, P5abc RNA thus serves as a molecular sensor to distinguish between the correct and misfolded ∆P5 ribozyme structures [35].

To prevent splicing and related reactions of the intron ribozyme during EMS analysis, we introduced mutations to eliminate the catalytic ability of the ribozyme without disturbing its 3D structure (Figure 1A). We substituted a G-C pair (corresponding to G264 and C311 in the parent *Tetrahymena* ribozyme) in the P7 element. This C-G pair is completely conserved among all group I introns and serves as the binding site for guanosine (G-site) [13,14]. In the mechanism of group I intron self-splicing, the G-site G-C pair directly recognizes an exogenous guanosine cofactor in the first step (cleavage of the 5′ splice site) [13]. The G-site also defines and activates the 3′ splice site by recognizing the terminal guanosine [9,10,11,14], which corresponds to G414 in the parent ribozyme and is shown as ωG in Figure 1A, in the second step. It has been shown that substitution of the G-site G-C pair to the C-G pair inactivates the ribozyme because this base pair substitution disrupts the guanosine recognition ability of the P7 element [14,36]. We prepared C264-G311 mutants of the site-2 (mut site-2) and site-3 (mut site-3) ribozymes to eliminate their guanosine-dependent splicing ability (Figure 1A).

In the presence of fluorophore-labeled P5abc RNA, EMS analysis was performed using precursors of the site-1, site-2, mut site-2, site-3, and mut site-3 ribozymes (Figure 4). In the absence of Mg^2+^ ions (Figure 4A) or ∆P5 ribozyme precursors (Lane 1 in Figure 4B,C), free P5abc RNA was only observed. In the presence of ∆P5 ribozyme precursors and 5 mM Mg^2+^ ions (Lanes 2–6 in Figure 4B), the amount of free P5abc RNA decreased and new bands corresponding to ∆P5 ribozyme/P5abc RNA complexes were observed. In the presence of site-1 and site-3 (and mut site-3) RNAs, most P5abc RNA formed a complex with ∆P5 ribozyme. In contrast, P5abc RNA formed complexes less efficiently with the precursor of site-2 and mut site-2 RNAs. Although complex formation of the site-2 and mut site-2 RNAs improved modestly with 15 mM Mg^2+^ ions, complex formation was still incomplete and less efficient than those of site-1 and site-3 RNAs (Figure 4C). These results suggest that the tertiary structure of ∆P5 ribozyme formed correctly in the site-1 precursor, while the ∆P5 ribozyme structure within the site-2 precursor may have a misfolded region. The folding problem of the site-1 precursor RNA may therefore occur at an exonic sequence, including the exon–intron junctions. This possibility is supported by structural prediction of the exon–intron junction at the 5′ splice site, where the correct P1 structure of the site-1 ribozyme is thermodynamically less favored than an alternative structure that preserves the secondary structure of the spinach aptamer (Figure 4D). An incorrect P1–P10 structure (Figure 4D-right) would inhibit the correct recognition of 5′ splice site but would not affect the complex formation with P5abc RNA. Currently, no possible alternative (misfolded) structure has been identified for site-2 precursor RNA through structural prediction.

### 3.4. Physical Dissection of Spinach RNA to Resolve Folding Problems of ∆P5 Ribozyme

**Figure 5:**
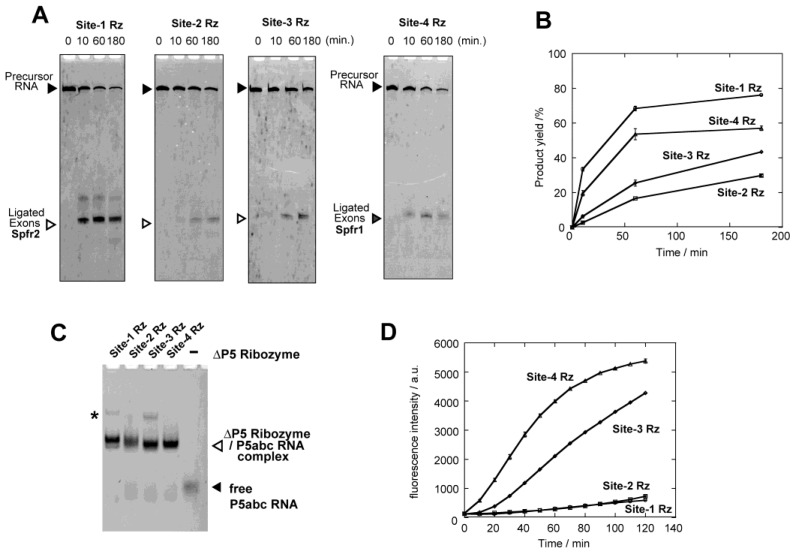
Characterization of the bimolecular ribozymes bearing fragments of spinach RNA as exonic sequences with no rigid secondary structures. (**A**) Denaturing polyacrylamide gel images of splicing reactions. (**B**) Time courses of the production of Spfr1 RNA or Spfr2 RNA as ligated exons. (**C**) EMS assay of ∆P5 ribozyme/P5abc RNA complexes with Spfr1 or Spfr2 RNA as flanking exons. Asterisk indicates weak bands, the identity of which is unknown. (**D**) Time-dependent increases in fluorescence of DFHBI captured by the bimolecular spinach RNA. Splicing reactions yield either Spfr1 RNA (from site-4 Rz) or Spfr2 RNA (from site-1, -2, and -3 Rzs) as ligated exons. Fluorescence was monitored with a 40 µL solution.

Although the structural basis of splicing deficiency of the site-2 ribozyme has not been elucidated, misfolding of the precursor RNAs must be involved. To solve the possible folding problem of the precursor RNAs, we physically dissected spinach RNA into two pieces (Spfr1 and Spfr2 in Figure 1B). The resulting fragments reconstitute the function of spinach RNA through bimolecular assembly. In the bimolecular spinach aptamer, site-1, site-2, and site-3 ribozymes were inserted in the Spfr2 RNA, whereas site-4 ribozyme was inserted into the Spfr1 RNA. The three precursors (site-1, -2, and -3) with Spfr2 exons were all active to yield Spfr2 RNA in the presence of P5abc RNA and 5 mM Mg^2+^ ions (Figure 5A). The extent of splicing reaction varied among the three precursor RNAs, among which the site-1 ribozyme with Spfr2 exons was more active than the site-2 and site-3 ribozymes (Figure 5B). In EMS analysis, P5abc RNA showed similarly retarded bands with four precursor RNAs (Figure 5C). This result suggests that possible folding problems in site-1 and site-2 with full-length spinach exons were resolved in the precursors with Spfr2 exons.

Encouraged by the results of splicing and EMS assays of ∆P5 ribozymes with Spfr2 exons, we monitored the increase in fluorescence that was produced by cotranscriptional splicing assisted by P5abc RNA followed by association of the spliced product (Spfr2 RNA) with Spfr1 RNA and DFHBI. Fluorescence measurement indicated that three precursor RNAs produced fluorescence of DFHBI–spinach RNA complex although their fluorescence varied (Figure 5D). The site-1 and site-2 RNAs, which showed no fluorescence in splicing with the full-length spinach exons with 5 mM Mg^2+^ ions (Figure 2 and Figure 3), exhibited weak but detectable emission (Figure 5D). On the other hand, the extent of emission was significantly different from the extent of the production of Spfr2 as ligated exons analyzed by denaturing PAGE (Figure 5B). One possible reason for the gap between splicing activity determined by PAGE and fluorescence of DFHBI–spinach RNA complex may be the release process of the ligated exons (Spfr2) from ∆P5 ribozyme, which was required for the bimolecular folding of spinach RNA. The release of the ligated exons from the excised intron requires disassembly of a 13-base pair P10 duplex between the two RNAs. The splicing reaction of the site-4 ribozyme with Spfr1 exons was also tested (Figure 5D). In the presence of P5abc RNA, the site-4 precursor efficiently produced Spfr1 RNA, which formed the functional bimolecular spinach RNA with Spfr2 RNA (Figure 5D).

### 3.5. Application of Bimolecular Spinach RNA to Analyze Engineered *Tetrahymena* Ribozymes

**Figure 6:**
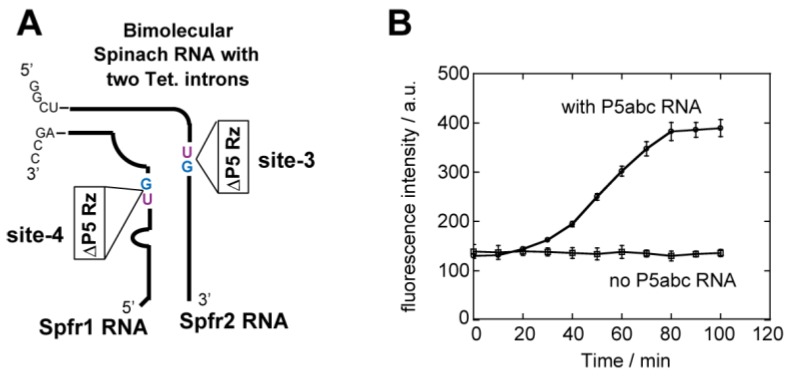
Reconstitution of the bimolecular spinach RNA through excision of two ∆P5 introns. (**A**) Schematic structure of the bimolecular spinach RNA, in which two fragments both contain ∆P5 ribozymes as introns. (**B**) Time-dependent increases in fluorescence of DFHBI captured by the bimolecular spinach RNA. Spfr1 RNA and Spfr2 RNA were both yielded as ligated exons. Fluorescence was monitored with a 40 µL solution.

Based on the observation that the site-3 and site-4 ribozymes spliced from Spfr1 and Spfr2 RNAs and the resulting ligated exons formed the bimolecular spinach aptamer (Figure 5), we inserted ∆P5 ribozymes into both Spfr1 and Spfr2 simultaneously to site-4 and site-3 in the respective RNA fragments (Figure 6A). A solution containing two precursor RNAs of Spfr1 and Spfr2 produced emission of the bimolecular spinach aptamer only when the two precursor RNAs performed splicing reactions. Florescence monitoring of the reaction solution containing the two precursor RNAs exhibited emission of the spinach–DFHBI complex in a P5abc-dependent manner (Figure 6B), indicating two ∆P5 ribozymes inserted into the Spfr1 and Spfr2 spliced to form functional bimolecular spinach RNA.

## 4. Discussion

In this study, we constructed a model system to analyze the self-splicing of the *Tetrahymena* group I intron, the exonic sequence of which, in the precursor, forms stable secondary and 3D structures in its product form. In this system, the formation of the ligated exons can be monitored readily by the fluorescence of the ligated exons (serving as an aptamer) complexed with the fluorophore, DFHBI. In the precursor RNAs with the full-length spinach aptamer as the exonic sequence, the efficiency of production of the functional ligated exons varied significantly among the four insertion sites of the intron ribozyme tested in this study. This difference was largely resolved in the truncated exonic sequences (Spfr1 and Spfr2), presumably because the truncated exonic sequences no longer formed a stable secondary structure. In the presence of the partner strand to reconstitute the bimolecular spinach aptamer (Spfr1 + Spfr2), the formation of the bimolecular spinach aptamer varied among precursor RNAs. In the site-1 and site-2 RNAs with Spfr2 exons, the partner RNA fragment (Spfr1) may perturb the catalytically active structure of the intron ribozyme or release of the ligated exons from the excised intron may proceed inefficiently. Use of spinach RNA aptamer as an exonic sequence of group I intron yielded a variable phenotype in self-splicing reactions according to the insertion position of the intron. Further studies of this model splicing system, especially from the viewpoint of interplay between two structured RNA elements (spinach aptamer as the exon and group I ribozyme as the intron), would provide valuable information that provides a better understanding of the self-splicing of group I introns.

The model splicing system constructed in this study is applicable to the development of molecular tools that control the production of functional RNAs in a post-transcriptional manner. In this study, we constructed a bimolecular spinach aptamer with two group I introns (Figure 6A). This system can be regarded as a simple model RNA system, in which two RNAs act cooperatively to conduct particular functions that depend on two intron splicing reactions. Self-splicing ribozymes are a promising new class of regulatory tools for synthetic RNA biology [37] because they can control two (or more) RNA processing reactions in a cooperative manner [38]. Our bimolecular spinach aptamer with two introns may serve as a model platform to develop novel splicing-based gene regulation systems.

## 5. Conclusions

In this study, we employed a fluorescent aptamer RNA (spinach RNA) as a model exonic sequence inserted by the *Tetrahymena* group I intron. The resulting system enables fluorescent monitoring of the self-splicing reaction of the *Tetrahymena* ribozyme. Although it needs to be further improved mainly through optimization of the insertion site of the intron ribozyme and its P1–P10 elements, this system can be applied in the real-time monitoring of self-splicing reactions not only in test tube but also in living cells. The strategy of this splicing monitoring system would also be applicable not only to other group I intron ribozymes but also to other classes of RNA-based splicing systems such as group II intron ribozymes and pre-mRNA introns excised by spliceosome.

## References

[1] Lilley D.M., Eckstein F. (2007). Ribozymes and RNA Catalysis.

[2] Elliott D., Ladomery M. (2016). Molecular Biology of RNA.

[3] Cech T.R. (1990). Nobel lecture. Self-splicing and enzymatic activity of an intervening sequence RNA from *Tetrahymena*. Biosci. Rep..

[4] Phizicky E.M., Greer C.L. (1993). Pre-tRNA splicing: Variation on a theme or exception to the rule?. Trends Biochem. Sci..

[5] Golden B.L. (2007). Group I introns: Biochemical and crystallographic characterization of the active site structure. Ribozymes and RNA Catalysis.

[6] Woodson S.A. (2005). Structure and assembly of group I introns. Curr. Opin. Struct. Biol..

[7] Vicens Q., Cech T.R. (2006). Atomic level architecture of group I introns revealed. Trends Biochem. Sci..

[8] Stahley M.R., Strobel S.A. (2006). RNA splicing: Group I intron crystal structures reveal the basis of splice site selection and metal ion catalysis. Curr. Opin. Struct. Biol..

[9] Adams P.L., Stahley M.R., Kosek A.B., Wang J., Strobel S.A. (2004). Crystal structure of a self-splicing group I intron with both exons. Nature.

[10] Golden B.L., Kim H., Chase E. (2005). Crystal structure of a phage *Twort* group I ribozyme-product complex. Nat. Struct. Mol. Biol..

[11] Guo F., Gooding A.R., Cech T.R. (2004). Structure of the *Tetrahymena* ribozyme: Base triple sandwich and metal ion at the active site. Mol. Cell.

[12] Weinstein L.B., Jones B.C., Cosstick R., Cech T.R. (1997). A second catalytic metal ion in group I ribozyme. Nature.

[13] Michel F., Hanna M., Green R., Bartel D.P., Szostak J.W. (1989). The guanosine binding site of the *Tetrahymena* ribozyme. Nature.

[14] Been M.D., Perrotta A.T. (1991). Group I intron self-splicing with adenosine: Evidence for a single nucleoside-binding site. Science.

[15] Haugen P., Simon D.M., Bhattacharya D. (2005). The natural history of group I introns. Trends Genet..

[16] Phylactou L.A., Darrah C., Wood M.J. (1998). Ribozyme-mediated *trans*-splicing of a trinucleotide repeat. Nat. Genet..

[17] Sullenger B.A., Cech T.R. (1994). Ribozyme-mediated repair of defective mRNA by targeted, trans-splicing. Nature.

[18] Jones J.T., Sullenger B.A. (1997). Evaluating and enhancing ribozyme reaction efficiency in mammalian cells. Nat. Biotechnol..

[19] Campbell T.B., Cech T.R. (1995). Identification of ribozymes within a ribozyme library that efficiently cleave a long substrate RNA. RNA.

[20] Dotson P.P., Hart J., Noe C., Testa S.M. (2012). Ribozyme-mediated *trans* insertion-splicing into target RNAs. Methods Mol. Biol..

[21] Amini Z.N., Olson K.E., Müller U.F. (2014). Spliceozymes: Ribozymes that remove introns from pre-mRNAs *in trans*. PLoS ONE.

[22] Olson K.E., Müller U.F. (2012). An in vivo selection method to optimize *trans*-splicing ribozymes. RNA.

[23] Satterwhite L.E., Yeates J.A., Lehman N. (2016). Group I intron internal guide sequence binding strength as a component of ribozyme network formation. Molecules.

[24] Paige J.S., Wu K.Y., Jaffrey S.R. (2011). RNA mimics of green fluorescent protein. Science.

[25] Warner K.D., Chen M.C., Song W., Strack R.L., Thorn A., Jaffrey S.R., Ferré-D'Amaré A.R. (2014). Structural basis for activity of highly efficient RNA mimics of green fluorescent protein. Nat. Struct. Mol. Biol..

[26] Huang H., Suslov N.B., Li N.S., Shelke S.A., Evans M.E., Koldobskaya Y., Rice P.A., Piccirilli J.A. (2014). A G-quadruplex-containing RNA activates fluorescence in a GFP-like fluorophore. Nat. Chem. Biol..

[27] Williams K.P., Fujimoto D.N., Inoue T. (1992). A region of group I introns that contains universally conserved residues but is not essential for self-splicing. Proc. Natl. Acad. Sci. USA.

[28] Ikawa Y., Moriyama S., Furuta H. (2008). Facile syntheses of BODIPY derivatives for fluorescent labeling of the 3′ and 5′ ends of RNAs. Anal. Biochem..

[29] Sargueil B., Tanner N.K. (1993). A shortened form of the *Tetrahymena thermophila* group I intron can catalyze the complete splicing reaction *in trans*. J. Mol. Biol..

[30] Ikawa Y., Yoshimura T., Hara H., Shiraishi H., Inoue T. (2002). Two conserved structural components, A-rich bulge and P4 XJ6/7 base-triples, in activating the group I ribozymes. Genes Cells.

[31] Donghi D., Schnabl J. (2011). Multiple roles of metal ions in large ribozymes. Met. Ions Life Sci..

[32] Joyce G.F., van der Horst G., Inoue T. (1989). Catalytic activity is retained in the *Tetrahymena* group I intron despite removal of the large extension of element P5. Nucleic Acids Res..

[33] Van der Horst G., Christian A., Inoue T. (1991). Reconstitution of a group I intron self-splicing reaction with an activator RNA. Proc. Natl. Acad. Sci. USA.

[34] Engelhardt M.A., Doherty E.A., Knitt D.S., Doudna J.A., Herschlag D. (2000). The P5abc peripheral element facilitates preorganization of the *Tetrahymen*a group I ribozyme for catalysis. Biochemistry.

[35] Johnson T.H., Tijerina P., Chadee A.B., Herschlag D., Russell R. (2005). Structural specificity conferred by a group I RNA peripheral element. Proc. Natl. Acad. Sci. USA.

[36] Couture S., Ellington A.D., Gerber A.S., Cherry J.M., Doudna J.A., Green R., Hanna M., Pace U., Rajagopal J., Szostak J.W. (1990). Mutational analysis of conserved nucleotides in a self-splicing group I intron. J. Mol. Biol..

[37] Che A.J., Knight T.F. (2010). Engineering a family of synthetic splicing ribozymes. Nucleic Acids Res..

[38] Tanaka T., Matsumura S., Furuta H., Ikawa Y. (2016). Tecto-GIRz: Engineered group I ribozyme the catalytic ability of which can be controlled by self-dimerization. ChemBioChem..

